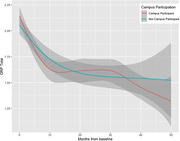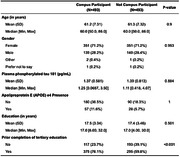# Impact of free later life formal university education on longitudinal modifiable risk factors, cognition and plasma phosphorylated tau 181

**DOI:** 10.1002/alz.090059

**Published:** 2025-01-09

**Authors:** Eddy Roccati, Aidan Bindoff, Alex Kitsos, Jane E Alty, Jessica M Collins, Anna E King, Kathleen Doherty, James C Vickers

**Affiliations:** ^1^ Wicking Dementia Research and Education Centre, University of Tasmania, Hobart, TAS Australia; ^2^ Royal Hobart Hospital, Hobart, TAS Australia

## Abstract

**Background:**

Previous research has focused on early‐life education to reduce dementia risk, yet there is great potential for enhancing cognitive reserve in later‐life through educational interventions, even for people with low early‐life educational attainment. In 2019, we launched ISLAND (Island Study Linking Ageing and Neurodegenerative Disease) Campus, offering free university study to participants, with flexible in‐person/online learning models removing educational, socioeconomic and geographical barriers. After four years, here we investigate our core hypothesis: that engagement in later life education leads to improvements in modifiable risk factors for dementia, cognition and plasma biomarkers.

**Method:**

ISLAND Campus participants were matched on age and gender to non‐Campus participants via propensity score method, with optimal matching based on logistic regression. Participants completed online surveys on background health, demographics, modifiable dementia risk via customised Dementia Risk Profile (DRP) and provided a blood sample for APOE genotyping and plasma phosphorylated‐tau (p‐tau) 181. Cognition was measured online via the validated Cambridge Neuropsychological Test Automated Battery Paired Associates Learning (PAL) and Spatial Working Memory (SWM) tasks. Impact of the opt‐in educational intervention was tested in R via ANCOVA.

**Result:**

Included were 986 participants (intervention = 493, control = 493), mean age 61.2 years, 71.2% female, 11.7 mean years of education, 23.9% APOE e4+. Intervention and control participants were similar on socioeconomic status, location of residence, p‐tau and APOE e4 presence, however intervention participants had significantly higher history of prior university study completion (76.1%) than controls (59.8%). Intervention participants enrolled in a variety of university degrees, the most common were Diploma of Family History (n = 103, 20.8%), Diploma of Arts (n = 74, 15.0%) and Diploma of Fine Arts (n = 52, 10.6%). Over four years of follow‐up, intervention participants significantly improved episodic memory (PAL) and their risk factor profiles as measured via the DRP (p <0.001), indicating a significant change towards lower dementia risk.

**Conclusion:**

We found free later‐life university education was associated with improvements in modifiable dementia risk factors over time and cognition. Intervention participants displayed significantly higher baseline education than control participants, therefore later life educational interventions should be targeted at individuals with lower baseline education.